# Relationships in couples treated with sperm donation - a national prospective follow-up study

**DOI:** 10.1186/1742-4755-11-62

**Published:** 2014-08-07

**Authors:** Gunilla Sydsjö, Agneta Skoog Svanberg, Marie Bladh, Claudia Lampic

**Affiliations:** 1 Obstetrics and Gynecology, Department of Clinical and Experimental Medicine, Faculty of Health Sciences, Linköping University, Linköping, Sweden; 2 Department of Women’s and Children’s Health, Uppsala University, Uppsala, Sweden; 3 Department of Neurobiology, Care Sciences and Society, Karolinska Institutet, Sweden; 4 Department of Gynecology and Obstetrics in Linköping, County Council of Östergötland, Linköping, Sweden

**Keywords:** Couples relationship, Donation, Infertility

## Abstract

**Background:**

Long-term follow-up on relationship quality in couples who use sperm donation is scarce. Therefore, this study aimed to analyse changes over time in satisfaction with relationship in heterosexual couples who were scheduled for treatment with sperm donation and IVF couples treated with their own gametes and to compare the two groups undergoing different treatment for infertility.

**Method:**

A prospective follow-up study in which data were collected twice on two groups; couples receiving sperm donation and IVF couples using their own gametes. The ENRICH instrument was used to gain information about the individuals’ subjective experience of their relationship at the time of acceptance for treatment and again 2–5 years later.

**Results:**

At the time of acceptance for treatment the men and women in the two groups assessed their relationships as being very solid on all dimensions and that there were no differences between the two groups. At the second assessment there was a decline in the satisfaction scores on the dimensions “Children and parenting” and “Egalitarian”, while an increase in scores was observed on “Conception of life” and “Conflict resolution” both for men and woman and also for the two groups. For the couples that had a successful treatment and gave birth to a child/children there was a decrease in satisfaction of the relation in the sperm donation group as well as in the group of couples having IVF with own gametes.

**Conclusion:**

In conclusion, the overall quality of relationship is stable in couples receiving donated sperm and does not differ from couples undergoing IVF-treatment with own gametes.

## Background

Studies on how heterosexual couples adjust to dealing with different types of infertility diagnoses and subsequent treatment are generally in agreement in that most couples appear to handle their relationship and adjustment well, both before and after treatment [1-5]. The couple might react to this new situation by developing an even stronger partner relationship or by experiencing some negative changes in their relationship.

For most men who, with their female partners, seek help for infertility and being given a male-factor diagnosis, it will be an unexpected and shocking experience. To be diagnosed to have an azoospermia and thus not being able to archive pregnancy or having another male factor diagnose with a very small chance to achieve pregnancy might draw attention to the couple’s positions, interaction and the balance in their dyad/relationship. For a man the fact that he is not able to produce children could be a threat to his manhood, self-esteem and mental wellbeing and as a consequence a risk to the relationship and future family constellations [6].

Men’s reaction to infertility because of a male-factor deficiency has been sparsely studied but reports from studies on men and women in IVF settings suggest that men and women react in similar manner when faced with an infertility problem [7], but do exhibit different coping strategies. When men learn that they are the ones who are infertile, they experience the same degree of low self-esteem, stigma, and depression as women who are infertile do. Men’s coping strategies differ though and this has been confirmed by Peterson et al. [8] who found that men in primary infertile IVF couples used coping strategies such as planned problem solving and distancing themselves from the problem [8]. Women show a different approach to cope with their infertility situation such as seeking social support and thus communicating their problem to others.

Couples who have not been able to get pregnant with own gametes but then manage to have a child seem to handle their relationship in a balanced way as well. In a 20 year long term follow up on 514 couples we found that the subjective opinion expressed by couples who had continued to stay together during that time was that their relationship continued to be good, whether they had become parents or not [3]. Noteworthy was also that the majority of IVF couples (90.8%) who had been treated 20 years prior to follow-up had added at least one biological or adopted child to the family during that time.

Very few studies have studied men selected because a clear medical indication for treatment with donated sperms such as azoospermia. The studies that have been made are characterized by a short follow up time. Furthermore, the aspect of becoming a parent by donated gametes or not is of interest but seldom taken into account in the interpretation of the analyses over time. Additionally the focus has been mostly on infertility in general and mainly on IVF treatment with own gametes.

The aim of the present study was twofold: to investigate relationship quality before and 2–5 years after sperm donation treatment among heterosexual couples and to compare relationship quality between couples receiving sperm donation treatment and couples receiving IVF-treatment with own gametes.

## Materials and methods

### Procedures

All couples who were to be given treatment were asked to participate in the “*The Swedish Study on Gamete Donation*” which is a prospective longitudinal study of donors and recipients of donated gametes. The multicentre study draws on subjects from all infertility clinics employing gamete donation in Sweden, clinics located at the University hospitals in Stockholm, Gothenburg, Uppsala, Umea, Linköping, Örebro, and Malmö. During the period 2005 to 2008 consecutive samples of couples starting donation treatment were approached regarding participation and asked to complete the ENRICH inventory. Exclusion criteria when enrolling in the study were inability to read and write the Swedish language.

### Index group

We only considered heterosexual couples accepted for treatment with donated gametes. For heterosexual sperm-receiving couples a total 330 individuals (165 couples) were eligible for participation, and of these 52 declined to participate, 26 were excluded and 7 did not respond, leaving 245 individuals. In this study we only report on those where *both* individuals in a couple have answered the ENRICH (Evaluating & Nurturing Relationship. Issues, Communication & Happiness) inventory at the first assessment and who were still living together at the second assessment of ENRICH, in total 208 individuals (104 couples). Of these, a total of 98 individuals (49 couples) have given complete answers for both the first and second assessment of the ENRICH inventory.

### Comparison group

In this national long-term follow up study we have used a comparison group of heterosexual couples using their own gametes and undergoing IVF-treatment during the same time period at the five university clinics in Linköping, Örebro, Uppsala, Gothenburg and Umeå. This group of couples, men and women, serves as a comparison/control group in different parts of the total study as well as used for different aims. The participating IVF clinics recruited a consecutive sample of 25–30 couples each the only exclusion criteria when enrolling in the study were inability to read and write the Swedish language. The reasons for choosing these clinics were that they were representative for the Sweden population in general.

In all a consecutive sample of 212 heterosexual couples (424 individuals) starting assisted reproduction were approached for study participation during the same time period.. All of these couples agreed to participate and individually completed a questionnaire asking for demographic data as well as the ENRICH inventory at the start of treatment. In seven couples, only one partner chose to participate, resulting in a total of 302 participants (71% response). Reasons for non-participation were: did not want to participate (n = 72) treatment discontinuation (n = 42), or not stated (n = 8).

For this study, we only report on the couples where both individuals answered the ENRICH inventory at the first assessment and who were still living together at the second assessment of ENRICH, in total 238 individuals. Of these, 122 individuals (61 couples) have given complete answers for both the first and second assessment of the ENRICH inventory.

### First assessment, time-point 1 (T1)

In general the couples received oral information about the purpose of the study from their doctor at their own University clinic. They were also given written information. The couples were asked to complete the ENRICH inventory separately at the clinic or, if they preferred, at home and then send it to the project team.

### Second assessment, time-point 2 (T2)

The second assessment with the ENRICH inventory was made two-five years after start of treatment. The couples received an information letter and the ENRICH inventory was sent by mail to the men and women separately.

The length of time between being accepted to the program and receiving treatment was in general around 18–24 months for sperm recipients and for IVF couples around 6–12 months with variation between clinics.

### Ethical consideration

The study was approved by the Regional Ethical Review Board in Linköping.

### Measures

#### ENRICH inventory

We used the Swedish version of the ENRICH marital inventory, originally created by Olson and co-workers [9], to study marital/partner relationship functions and dynamics. The instrument provides scores of each partner’s evaluation of their relationship as they assessed their present relationship in 10 categories comprising 10 items each. The ENRICH different categories can be described as follows:

*Personality Issues*: Examines an individual's satisfaction with his or her partner's behaviours.

*Communication*: Is concerned with an individual's feelings and attitudes toward communication in the marriage. Items focus on the level of comfort felt by the respondent sharing and receiving emotional and cognitive information from the partner.

*Conflict Resolution*: Assesses the partner's perception of the existence and resolution of conflict in the relationship. Items focus on how openly issues are recognized and resolved, as well as the strategies used to end arguments.

*Financial Management*: Focuses on attitudes and concerns about the way economic issues are managed within the marriage. Items assess spending patterns and the manner in which financial decisions are made.

*Leisure activities*: Assesses preferences for spending free time and leisure time. Items reflect social vs. personal activities, shared vs. individual preferences, and expectations about spending leisure time as a couple.

*Sexual Relationship*: Examines the partner's feelings about the affectionate and sexual relationship. Items reflect attitudes about sexual issues, sexual behaviour, and sexual fidelity.

*Children and parenting*: Assesses attitudes and feelings about having and raising children. Items focus on decisions regarding discipline, goals for the children, and the supposed impact of children on the couple’s relationship.

*Family and Friends*: Assesses feelings and concerns about relationships with relatives, in-laws, and friends. Items reflect expectations for and comfort with spending time with family and friends.

*Egalitarian Roles*: Focuses on an individual's feelings and attitudes about various marital and family roles. Items reflect occupational, household, sex, and parental roles. High scores indicate a preference for more egalitarian roles.

*Conception of Life*: Examines the meaning of values, religious beliefs and practice, and conception of life within the marriage.

Each category scale can vary between 10 and 50 points, 50 points being the most positive outcome. There are six alternatives for each item ranging from “in total agreement” to “do not agree at all”. The summed category scale scores provide a global assessment of marital satisfaction varying between 100 and 500 points.

In addition, we used the Positive Couple Agreement (PCA) scale, which was derived from the ENRICH subscales. The PCA scale score is obtained by measuring the couple’s agreement in describing their relationship in positive terms on each question for each scale. This results in a measurement ranging from 0% to 100% agreement, depending on the number of positive agreements and the total number of questions in each subscale.

The ENRICH subscales have shown good internal consistency (alpha, range = .69-97) and test-retest reliability (r_tt_ , range = .65-.94) as well as content and construct validity ([9]). The discriminate and concurrent validities of these scales have been established [10]). The Swedish version of the inventory has been evaluated [11], whereby the reliability and the validity of the instrument have been established to be satisfactory. A quality check of the ENRICH inventory for each group in this study revealed that Cronbach’s alpha for the total score of ENRICH was 0.844 for sperm recipients and 0.879 for couples treated with traditional IVF at first assessment. For evaluation of drop-outs in this present study a cut-off value on the total score below 402 was used, which equals the 25^th^ percentile.

### Clinical practice during the study period

The university clinics that perform sperm donation treatment in Sweden all follow the same guidelines, those set by The National Board of Health and Welfare in Sweden. Consequently all couples seeking treatment with donor sperm have to be interviewed and be assessed both medically and psychologically. The couples should also be able to show that they are in a stable relationship defined as having a relationship for two years or more, living together and also that they both understand the treatment procedures and are willing to disclose to the child to be that he or she can, in the future, have access to the donor’s identity. For couples treated with their own gametes the couples should have a stable relationship as described above. The number of ART treatments such as insemination with donated sperms and or IVF with donated sperms or the couple’s own gametes differ from clinic to clinic depending on local healthcare policies – but approximately 2–3 insemination and or two to three IVF treatment cycles are what are offered free of charge i.e. included in the ordinary health security program as specified by Swedish law.

### Statistics

The ENRICH scores (i.e. the ten factors as well as the total scores on both occasions) for the study group were tested for normality by use of the Kolmogorov-Smirnov test. The data were also examined visually by scatter-plots to identify possible extreme values. As the assumption of normality could not be met in all of the studied variables, we chose to use a non-parametric approach when analysing the data. The Mann–Whitney U test was used to examine differences between the two study groups, “no children through treatment” and “children through treatment”. To evaluate change in scores over time the Wilcoxon paired signed ranks test was performed. In addition, chi-square test was used to analyse group differences in socio-demographic variables. All statistical analyses were performed using IBM SPSS, version 19.0 (IBM SPSS Inc., Armonk, NY). A p-value <0.05 was considered statistically significant.

## Results

The demographic data for the participating couples is displayed in Table 1. No differences were detected between the two groups, apart from the variable indicating whether the treatment had resulted in a child. Sperm recipients were more likely to have children after treatment compared to couples who had undergone traditional IVF treatment. However, when comparing men and women in the two treatment groups, men were older and had a lower level of education compared to the women.

**Table 1:** Demographic data for women and men participating in the study

		**Sperm recipients (n = 208)**				**Traditional IVF (n = 238)**				**Women**	**Men**		
		**Women**		**Men**		**Women**		**Men**				**Sperm**	**IVF**
		**n**	**%**	**n**	**%**	**n**	**%**	**n**	**%**	p-value*	p-value*	p-value*	p-value*
Age	≤30	34	32.7	21	20.2	41	34.5	29	34.4	0.781	0.456	0.041	0.088
	>30	70	67.3	83	79.8	78	65.5	90	75.6				
Education	Elementary	2	1.9	7	6.8	4	3.4	10	8.5	0.792	0.653	0.005	0.040
	High school	39	37.9	56	54.4	46	38.7	57	48.3				
	University	62	60.2	40	38.8	69	58.0	51	43.2				
Biological children	No	92	88.5	99	95.2	107	89.2	109	90.8	0.867	0.206	0.076	0.884
	Yes	12	11.5	5	4.5	13	10.8	11	9.2				
Adoptive children	No	104	100.0	103	99.0	120	100.0	120	100.0	-	0.464	-	-
	Yes			1	1.0			0	0.0				
Step children	No	100	96.2	100	96.2	117	97.5	113	94.2	0.564	0.492	1.000	0.196
	Yes	4	3.8	4	3.8	3	2.5	7	5.8				
Child after treatment**	No	22	31.8	-	-	48	53.9	-	-	0.006	-	-	-
	Yes	47	68.0	-	-	41	46.1	-	-				

^*^Pearson’s Chi-square test. If cell count is below 5 Fisher’s exact test was used.

^**^Child after treatment is calculated according questionnaire at E3.

The first assessment at acceptance for treatment shows that men and women in the different groups assess their relations as very solid on all dimensions and there are no differences between to two groups (Table 2).

**Table 2:** The couples’ assessment of their relationship at acceptance for infertility treatment

	Sperm recipients* (n = 208)		Traditional IVF* (n = 238)		Sperm vs. IVF*	
	**Woman mean/SD**	**Man mean/SD**	**Woman mean/SD**	**Man mean/SD**	**Woman p-value**	**Man p-value**
Personality	43.8/4.3	42.2/5.0	43.4/4.6	41.7/5.1	0.641	0.413
Sexual	43.2/3.2	43.6/6.2	43.2/3.3	43.0/3.6	0.473	0.318
Children	44.1/3.0	43.6/3.6	43.8/3.9	43.7/3.4	0.651	0.452
Family	44.0/4.0	43.1/4.2	44.5/3.9	43.2/5.1	0.783	0.943
Egalitarian	40.7/3.6	41.4/3.4	40.5/3.6	41.1/3.6	0.642	0.071
Conception	40.4/3.2	40.0/3.5	39.9/3.5	39.1/4.0	0.919	0.769
Communication	43.7/4.9	43.3/4.7	43.4/4.8	42.5/5.1	0.781	0.907
Conflict	41.0/4.0	40.0/5.0	40.4/5.4	39.3/5.9	0.366	0.546
Financial	43.0/4.1	42.4/4.3	42.9/4.0	42.2/4.6	0.613	0.535
Leisure	40.9/4.7	39.4/5.3	40.6/4.5	38.1/5.7	0.170	0.026
Total	424.9/24.7	419.1/30.22	422.4/28.0	413.9/33.4	0.713	0.287

*Mann–Whitney U-test.

At the second assessment there was a decline in the scores on the dimensions “Children and parenting” and “Egalitarian” while an increase in scores was detected on the dimensions “Conception of life” and “Conflict resolution” both for men and woman within the sperm recipient group as well as among the couples using own gametes. Moreover a significant drop in the total score was seen when comparing the assessments at acceptance and 2–5 years after treatment start (Tables 3 and 4). The Positive Couple Agreement scores were also lowered in the two groups on most of the dimensions (Table 5) indicating a lower concurrence within dimensions between the individuals in the couples.

**Table 3:** The couples’ assessment of their relationship two–five years after treatment

	Sperm recipients* (n = 98)		Traditional IVF* (n = 122)		Sperm vs. IVF*	
	**Woman mean/SD**	**Man mean/SD**	**Woman mean/SD**	**Man mean/SD**	**Woman p-value**	**Man p-value**
Personality	42.5/5.9	41.8/5.6	41.9/5.8	42.2/8.5	0.523	0.557
Sexual	41.9/6.2	42.8/9.4	41.5/9.2	41.8/10.2	0.366	0.256
Children	39.6/5.1	38.6/6.3	38.4/6.1	37.3/6.6	0.199	0.193
Family	43.1/4.5	43.0/4.8	42.2/5.3	42.6/6.7	0.457	0.478
Egalitarian	40.4/5.6	38.0/6.0	38.4/5.8	36.8/6.7	0.076	0.443
Conception	42.8/5.8	44.0/10.7	42.2/6.0	41.4/6.1	0.544	0.257
Communication	41.9/4.3	42.4/4.3	40.6/5.1	40.2/5.5	0.240	0.029
Conflict	43.2/5.0	42.9/5.3	42.2/4.5	42.2/6.5	0.136	0.240
Financial	39.2/4.6	40.9/4.3	38.5/4.4	40.0/4.2	0.402	0.198
Leisure	38.7/4.0	38.6/4.9	37.8/4.7	37.7/4.2	0.306	0.191
Total	413.2/37.8	413.14/41.1	403.5/41.9	402.1/46.3	0.270	0.203

*Mann–Whitney U-test.

**Table 4:** Test for difference on the ENRICH scores for each subscale comparing measurements before treatment and two-five years after treatment/childbirth

	Sperm recipients*		Traditional IVF*	
	**Woman**	**Man**	**Woman**	**Man**
Personality	0.174	0.540	0.001	0.560
Sexual	0.543	0.689	0.619	0.114
Children	<0.001	0.013	<0.001	<0.001
Family	0.267	0.707	0.006	0.073
Egalitarian	0.019	0.028	<0.001	0.001
Conception	<0.001	0.001	0.006	0.004
Communication	0.007	0.155	0.003	0.005
Conflict	0.008	0.004	0.001	0.001
Financial	0.557	0.709	0.429	0.553
Leisure	0.168	0.113	0.001	0.027
Total	0.002	0.066	<0.001	0.002

*Wilcoxon Paired Signed Ranks Test.

**Table 5:** Test for difference on PCA-scores between measurements before treatment and two–five years after treatment/childbirth

	**Sperm recipients**		**Traditional IVF**		Sperm recipients p-value*	Traditional IVF p-value*
	**T1**	**T2**	**T1**	**T2**		
Personality	67.2/20.3	62.4/20.0	65.4/19.9	60.8/23.8	0.012	0.001
Sexual	85.2/15.8	70.2/26.5	84.5/17.3	65.2/27.4	<0.001	<0.001
Children	77.3/12.7	69.4/19.2	75.2/17.1	61.0/23.4	<0.001	<0.001
Family	75.2/16.1	72.9/19.9	75.5/17.9	69.3/19.6	0.737	0.002
Egalitarian	67.3/15.0	62.2/19.1	64.5/15.7	57.2/20.4	0.002	<0.001
Conception	68.8/11.5	63.3/15.7	65.2/15.2	57.9/17.7	<0.001	0.001
Communication	74.5/20.5	64.5/28.2	72.7/21.1	60.3/27.6	<0.001	<0.001
Conflict	60.9/20.4	73.9/17.4	57.6/21.9	44.9/27.2	<0.001	<0.001
Financial	72.7/16.3	73.9/17.4	69.3/18.5	67.2/24.2	0.243	<0.152
Leisure	60.3/22.5	54.9/24.0	57.6/21.7	45.2/26.1	0.004	<0.001

*Wilcoxon Paired Signed Ranks Test.

Differences found by comparing child/no child within each group (i.e. sperm recipients and traditional IVF) were minimal. Actually, the only difference found was at the second assessment of the ENRICH inventory among individuals treated with traditional IVF. Women treated with traditional IVF who had become mothers during the study period scored lower on the ENRICH total score compared to those who had not become mothers (p = 0.013) Table 6. In Table 7, where data have been stratified into two groups according to child/no child after treatment, no differences were revealed when comparing the total scores at first and second assessment of the ENRICH scores between the two groups (sperm recipients and traditional IVF).

**Table 6:** Test for difference on the ENRICH scores for each subscale comparing measurements before treatment and two-five years after treatment/childbirth reported by type of treatment and child/no child after treatment

		**Sperm recipients**			**Traditional IVF**		
		**No child after treatment**	**Child after treatment**	p-value*	**No child after treatment**	**Child after treatment**	p-value*
Woman	Total at T1^1^	425.5/28.2	428.3/25.3	0.792	421.4/30.7	422.0/28.4	0.856
	Total at T2^2^	417.8/36.3	406.2/44.2	0.294	418.1/36.9	393.3/40.6	0.013
Man	Total at T1^1^	413.3/31.8	424.4/30.7	0.241	413.4/35.1	416.6/32.6	0.717
	Total at T2^2^	405.6/35.6	411.9/45.8	0.548	410.3/49.7	395.2/44.2	0.301

*Mann–Whitney U-test.

^1^First assessment, at start of treatment.

^2^Second assessment, 2–5 years after start of treatment.

**Table 7:** Test for difference on the ENRICH scores for each subscale comparing measurements before treatment and two-five years after treatment/childbirth reported by child/no child after treatment and type of treatment

		**No child after treatment (n = 54)**			**Child after treatment (n = 91)**		
		**Sperm recipients**	**Traditional IVF**	p-value*	**Sperm recipients**	**Traditional IVF**	p-value*
Woman	Total at T1^1^	425.5/28.2	421.4/30.7	0.344	428.3/25.3	422.0/28.4	0.970
	Total at T2^2^	417.8/36.3	418.1/36.9	0.166	406.2/44.2	393.3/40.6	0.876
Man	Total at T1^1^	413.3/31.8	413.4/35.1	0.690	424.4/30.7	416.6/32.6	0.334
	Total at T2^2^	405.6/35.6	410.3/49.7	0.949	411.9/45.8	395.2/44.2	0.192

^*^Mann–Whitney U-test.

^1^First assessment, at after start of treatment.

^2^Second assessment, 2–5 years after start of treatment.

Twenty-five percent of the men and women in this study had total Enrich scores below 402 so the vast majority (75%) had a much higher total score.

### Analysis of attrition

We performed an analysis on the couples that had dropped out after the first assessment and found that generally the rate of drop outs was higher among those who had a total ENRICH i.e. >402 compared to those with a low score < 402 (45% vs. 38%) at the first assessment.

## Discussion

Heterosexual couples that had been treated with sperm donation expressed satisfaction with their relationship two to five years after treatment. For the couples that had a successful treatment and gave birth to a child/children there was a decrease in the assessment of satisfaction of the relation in the sperm donation group as well as in the group of couples having IVF with own gametes. This decline should not be seen as alarming since this kind of change follows a trend that can be seen in IVF couples, lesbian couples as well as spontaneous conceiving couples in general [1-3,12].

Infertile men in the couples that were treated with donated sperm viewed their relationship as being stable throughout the study and it was observed that having children or being childless after treatment did not have a negative impact on their relationships. The selection process and the recommendation in Sweden that couples receiving donated games should have a stable relationship and also that the couples should be found healthy both mental and somatically at acceptance for treatment might also influence the relationship as well as the results in this study. The couples /individuals are not hampered with worries about illness and mental disturbances or an unequal relationship that could be the case if one of the spouses were ill.

The main strength of the present study is that this is a national cohort study covering all the 7 IVF centres performing treatment with donated sperm. The comparison group has been followed in the same manner during the same time period and circumstances. We have a good participation rate from the beginning of the study. Indications and treatments are fairly equal at the different centres indicating that we have a representative group of men and women treated for male factor diagnosis as well as for traditional IVF. The ENRICH is a useful inventory that measures different dimension of importance when assessing a relationship. The instrument has been used in previous studies on IVF couples and controls, which gives us an opportunity to compare different groups and development over time [1-3]. One limitation in this study is our inability to follow men and women from different origins who live in Sweden but could not read and write Swedish, this restricts the study’s generalizability. As in other long-term studies, attrition is also a concern for the generalizability of the results as well as for clinical implications. Is there a risk that study participants constitute couples who are keen to present acceptable and solid relationships, i.e. a social desirability effect, or do the results correctly reflect men and women undergoing infertility treatment have a stable relationship and, as shown earlier [1-3,5], that infertility may strengthen the relationship for some couples? There are also factors affecting the relationship that are hard to control for when researching relationship development over time e.g. illness, problems at work, child health, wishing for a sibling, trying to get pregnant again deciding on new treatments or adoption. These life events and social factors can all have an influence on the couples’ relationship.

To be infertile is a multidimensional stressor that has an impact on the individual as well as on the couple’s life both in the short and long term. It is well established that sustaining a long term relationship requires of the couple that both parties can communicate with one another. In addition, infertile couples can be faced with questions from family and friends that often arise when a couple does not have children. We suspect that a majority of the couples have a limited number of people, relatives and friends with whom they are able to share the reasons for being unable to conceive. Isaksson et al. [13] found that men in sperm-receiving couples talked to family and friends less often about their donation treatment in comparison with women who received treatment with donated oocytes or sperm [13]. When the factor preventing conception is so clearly a male factor, so that the only way for the woman to get pregnant is to use donated sperm, emotional reactions often arise that may be difficult for the couple to deal with. This also makes it more difficult for them to communicate with one another. If male infertility is viewed as a sensitive subject then the couple may be more likely to keep the reason for being unable to conceive a secret from relatives and significant others.

Keeping infertility a secret could also strengthen the couple’s relationship since they can discuss this subject only with each other. Then they have to engage in a decision process before they decide that sperm donation is the treatment they must elect, so they must agree on this decision. IVF couples treated with their own gametes may find it easier to make the decision to agree on IVF treatment and the waiting time before treatment could also be much shorter. The research by Pasch et al. [14] on marital quality in ART couples showed that when the spouses both were involved in the infertility treatment process the effects on the relationship were positive [14]. In a Finnish study by Repokari et al. [15] on marital relationships in ART couples (using their own gametes) and controls the results at the one year follow-up showed that the couples who had experienced involuntary childlessness were more resistant to negative psychosocial stressors compared to the control couples who were able to spontaneously conceive [15]. They also reported that they had a good and stable marital relationship throughout the treatment process. Research from Denmark [5,16] reported positive effects on marriage as a result of infertility experiences. These couples were IVF-couples using their own gametes after both successful and unsuccessful treatments. Peterson et al. [8] have also studied stress induced by infertility and found that both men and women used “plan full problem solving” as a coping mechanism and that might be an explanation of their ability to maintain a good and balanced relationship in that that they developed stronger feelings of being part of a joint effort to seeking a solution for the infertility [8].

The literature demonstrates that sexuality can be seriously affected by infertility and its treatment and that sexual problems might put pressure on the relationship of infertile couples. It is also evident that the sexuality of infertile men and women might be influenced by their partner’s reactions to the diagnosis of infertility [17]. We found no alarming decrease in satisfaction with the sexual relationship either in men or in women, a finding also in line with Repokari et al. [15] findings and results from other studies on the long-term relational effects on IVF couples [1-3].

The result in this study on the good positive agreement within the couples is an indicator that the couples can make decisions and handle stressful events such as infertility treatment whether based on using donated gametes or the couple’s own gametes. This is also in line with Blake et al. 2012 who have followed ART families, i.e. donor insemination, egg donation and surrogacy over 10 years and found that only a minority of couples divorced/separated and that the different family types did not differ substantially in terms of mothers' or fathers' perceptions of marital quality [18]. In a recent study on oocyte recipient couples we have show that both men and women who have been through a oocyte gamete treating program are satisfied and stable in their opinion about their relationship [19]. This was true for couples that have conceived and have had a child/children as well for the couples who were childless after treatment [19].

In the future, we need to have more research on the long-term effects on marriage and separations in families created with the help of ART techniques with donated gametes. We have a grey-area of couples that are not willing to be part of research, or drop out of research, and this is becoming a problem since we therefore have difficulties to interpret the effect of gamete donation for women and men and the future child. Do couples who decline participation in psychosocial research studies as this one have troubles in their relationship affecting them as individuals and parents, and also affecting the child in the future that needs extra support or awareness or are they the ones that have become parents and are happy ever after?

In conclusion, the present results indicate that the overall quality of relationship is stable in couples receiving donated sperm and does not differ from couples undergoing IVF-treatment with own gametes. However, for evaluation of our and others’ positive results we need more national register studies. Knowledge on family constructions and development is of importance for the politicians and the medical society in order to facility treatment and develop treatment suitable for individuals that have difficulties to achieve pregnancy without medical interventions.

## Competing interests

The authors declare that they have no competing interests.

## Authors' contributions

GS, ASS and CL planned and designed the study. GS, ASS, MB and CL contributed to the acquisition of data. GS, ASS, MB and CL analyzed the data and GS was primarily responsible for writing the paper. All authors were involved in drafting/revising of the paper and approved the final version of the manuscript for submission.
